# microRNAs as biomarkers of risk of major adverse cardiovascular events in atrial fibrillation

**DOI:** 10.3389/fcvm.2023.1135127

**Published:** 2023-02-21

**Authors:** Ascensión M. de los Reyes-García, Laura Zapata-Martínez, Sonia Águila, María L. Lozano, Constantino Martínez, Rocío González-Conejero

**Affiliations:** Servicio de Hematología y Oncología Médica, Hospital General Universitario Morales Meseguer, Centro Regional de Hemodonación, Universidad de Murcia IMIB Pascual Parrilla, Murcia, Spain

**Keywords:** mature microRNAs, polymorphisms, atrial fibrillation, thrombosis, inflammation, therapy

## Abstract

Atrial fibrillation is a complex and multifactorial disease. Although prophylactic anticoagulation has great benefits in avoiding comorbidities, adverse cardiovascular events still occur and thus in recent decades, many resources have been invested in the identification of useful markers in the prevention of the risk of MACE in these patients. As such, microRNAs, that are small non-coding RNAs whose function is to regulate gene expression post-transcriptionally, have a relevant role in the development of MACE. miRNAs, have been investigated for many years as potential non-invasive biomarkers of several diseases. Different studies have shown their utility in the diagnosis and prognosis of cardiovascular diseases. In particular, some studies have associated the presence of certain miRNAs in plasma with the development of MACE in AF. Despite these results, there are still many efforts to be done to allow the clinical use of miRNAs. The lack of standardization concerning the methodology in purifying and detecting miRNAs, still provides contradictory results. miRNAs also have a functional impact in MACE in AF through the dysregulation of immunothrombosis. Indeed, miRNAs may be a link between MACE and inflammation, through the regulation of neutrophil extracellular traps that are a key element in the establishment and evolution of thrombotic events. The use of miRNAs as therapy against thromboinflammatory processes should also be a future approach to avoid the occurrence of MACE in atrial fibrillation.

## 1. Introduction

From the epidemiological point of view, atrial fibrillation (AF) is the most prevalent arrhythmia and constitutes a social and healthcare issue, since its frequency doubles every decade after the age of 50 (1). In addition, this incidence continues to increase and is expected to double in the next three decades (2) and carries an inherent and independent risk of cardiovascular events with a fatal outcome (3, 4). In addition to these complications, AF-related outcomes such as depression, cognitive decline, or heart failure (2020 ESC Guideline) also provoke an impaired quality of life. Prophylactic anticoagulation has shown a clear benefit to prevent major adverse cardiovascular events (MACEs) in AF, however a residual risk for mortality has been reported between 4.4 and 7%/yr, with the majority of these deaths being related to MACE (5, 6).

**FIGURE 1:**
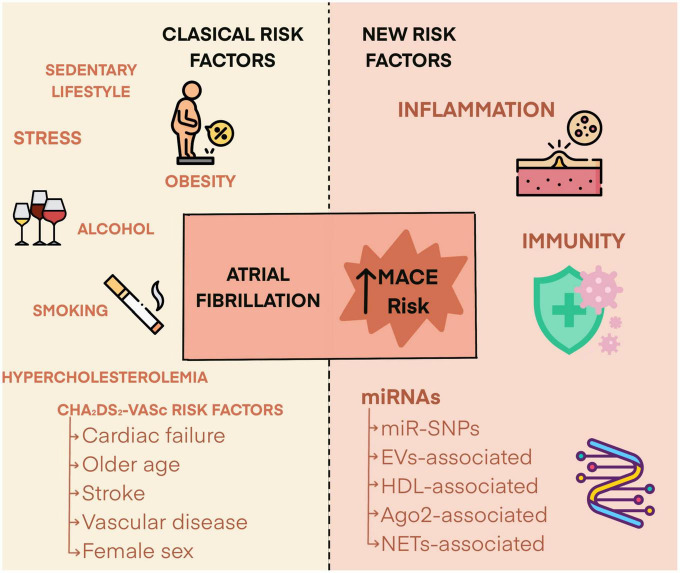
Risk factors determining the risk of major adverse cardiovascular events in atrial fibrillation patients.

For these reasons, and taking into account that a multitude of factors of a very diverse nature participate in the etiology of AF and that AF rarely occurs as an isolated phenomenon, the search for clinically useful markers in the prediction of MACE risk is still ongoing (Figure 1). Thus, markers for the onset and development of AF, as well as risk predictors for MACE, have been identified either from prospective and randomized clinical trials or from studies that, in any case, have recruited a large number of patients. Among them, parameters derived from cardiac and renal functions, and coagulation have helped in the understanding of the pathophysiological pathways leading to AF as well as in the forecast of risk for MACE (7, 8). More recently, inflammation began to be considered as a new factor that plays an important role not only in the onset of AF, but also in the AF-related MACE. Among the processes that sustain the connection between inflammation and MACE in AF, it has been suggested that activation of immune cells (monocytes, macrophages, lymphocytes), through proinflammatory cytokines secretion, promotes endothelial dysfunction and platelet activation (9).

## 2. Risk stratification in AF, a multisystem disease

All these mentioned markers are not mutually exclusive, but as it has been defined for other pathologies. The implementation of a multimarker strategy in AF has been pointed out as an optimal issue to ensure patient management. Thus, recent studies have assessed the superiority of multimarker strategies in risk stratification in large populations of patients. In the most recent clinical trial, Pol et al. have reported results from a sub-cohort from ARISTOTLE (Apixaban for Reduction in Stroke and Other Thromboembolic Events in Atrial Fibrillation) with a validation cohort from RE-LY study (10). Plasma samples were randomized and up to 268 biomarkers were analyzed. After almost a 2-year follow-up and a Cox analysis adjusted first in the identification cohort and later in the validation cohort, the authors confirmed that 10 biomarkers (involved in cardiac and renal functions, oxidative stress, inflammatory cytokines, calcium balance, fibrinolysis, and apoptosis) had the highest association with cardiovascular death (10).

These new insights indicate that AF should be considered as a multisystem disease (11). Indeed, the most recent guideline of ESC for the diagnosis and management of AF, proposes an integrated management team of different specialists to support the diverse patients’ needs (ESC GUIDELINE 2020). In addition, a recent meta-analysis, the PROSPERO clinical trial, highlights the permanent interest in refining risk scores to accurately categorize patients with AF. This systematic review of multiple risk scores confirmed a positive trend (which needs to be calibrated and externally validated) of improved risk prediction favorable to the newer scales compared to the more widely used CHADS_2_ and CHA_2_DS_2_-VASc (12). Taken together, these observations support that the identification of biomarkers that are predictors for risk of MACE should be extended to other organs and systems to achieve the best clinical follow-up in patients with AF. In this review, we will describe the data related to microRNAs (miRNAs), as next-generation biomarkers in the prediction of MACE in patients with AF.

## 3. Overview of the mechanisms of action of miRNAs

miRNAs are small endogenous non-coding RNAs whose function is to regulate the expression levels of their target genes, a process that is carried out by partial complementarity of miRNAs with bases located mainly in the 3′ untranslated region (3′UTR) of the target (13). They are involved nearly in all biological processes, from cell proliferation to apoptosis (14). Just in mammals, more than 50% of genes are regulated by miRNAs (15), therefore miRNAs are involved in practically all cellular processes (16).

The best known mechanism of action of miRNAs revolves around the complementarity of bases in their region in 5′ known as “seed” (around 6–8 nucleotides) with the corresponding bases of its target mRNA at the 3′UTR. In case of a perfect and full base complementary between miRNA and its target, the result will be the degradation of the mRNA and, if the pairing is imperfect the consequence will be the translation blockade that can happen with or without degradation of mRNA (17, 18). However, there have been several studies proving that miRNA/mRNA binding also occurs in the 5′UTR region (19) and even in some genes, in their promoter region (20, 21). Also, a gene (target) can have several binding sites for the same miRNA, leading to a more efficient inhibition. Furthermore, a single miRNA can regulate multiple target genes, being able to control several points of a pathway at the same time (22). There are diverse factors modulating the biogenesis or function of miRNAs, such as miR-SNPs, single nucleotide polymorphisms in genes coding for miRNAs or in their targets, that can modify the binding between miRNA and mRNA, and therefore have subsequent consequences on gene expression and disease risk (23).

## 4. miRNAs as biomarkers of disease

In 2004, miRNAs were observed in blood for the first time (24). miRNAs are highly stable in the body fluids, despite the RNAses in the environment (25), consequently, they have been gaining interest as a great non-invasive biomarker for several pathologies, especially in cancer (26), but also in cardiovascular diseases (CVD) (27, 28). Along with being easily accessible in blood, urine and other fluids, it has also been described that miRNA levels vary with the progress of the disease and even change with the evolution of the therapy (29, 30). Lawrie et al. performed the first study to measure miRNA levels in serum, and they found an association between the circulating miR-21 levels and the relapse-free survival in patients with diffuse large B-cell lymphoma (31). Later, several other studies demonstrated the potential of plasma miRNAs as biomarkers, especially at first in different types of cancers (32–34), and lately in CVD (35–37). Classically, miRNAs have been related to the pathophysiology of AF in relation to the atrial remodeling process, particularly, with the initiation and maintenance of AF. Abnormal levels of miRNAs in cardiac cells or, even, in blood can promote AF *via* atrial remodeling (38–40) data demonstrating a role for miRNAs in the pathophysiology of AF.

In relation with the identification of miRNAs as biomarkers of CV events, some reports have identified profiles of circulating miRNAs in patients with acute myocardial infarction (41), acute coronary syndrome (42) or coronary artery disease (43). In turn, the relationship between levels of miRNAs and the AF onset, the clinical management of patients, the adequacy of thromboprophylaxis or the severity of the disease have been extensively described by various groups (11, 44, 45), so in this review we will refer to the data that support a connection between miRNAs and the occurrence of MACE in patients with AF.

## 5. Circulating miRNAs and risk for MACE in AF

The prognostic role of MACE of circulating miRNAs in AF patients has only been explored recently. In a pilot study, our group analyzed the expression of 179 miRNAs in plasma of 9 AF patients who had cardiovascular events *vs*. 10 AF patients who did not have such events, respectively. The levels of candidate miRNAs that were identified in the discovery cohort (miR-22-3p, miR-107, and miR-146a-5p) were further validated in plasma from 166 patients of a validation cohort (46). A strength of this study was that patients were followed for almost 8 years, looking for an association of MACE registered in that period with the plasma levels of miRNAs at the diagnosis of AF (46). Thus, our results showed that patients with the highest risk of MACE had significantly higher levels of miR-22-3p and miR-107 and lower levels of miR-146a-5p. Furthermore, the inclusion of miR-107 and miR-146a-5p levels in the 2MACE score significantly improved the predictive ability of MACE compared to the original score (46, 47). Whether the inclusion of these (and/or other) miRNAs is a useful strategy to aid in the management and when making decisions about risk stratification of steadily anticoagulated patients with AF needs further confirmation in larger prospective studies.

In a similar exploratory study, Kiyosawa el al. analyzed the potential of 179 circulating miRNAs to correlate with CHA_2_DS_2_-VASc score. The ultimate goal of this study was to categorize patients with paroxysmal- (*N* = 22), persistent- (*N* = 10), and permanent- (*N* = 1) AF that needed performing catheter ablation 6 months after enrollment in accordance with their risk for stroke (48). Their results showed that 11 miRNAs were simultaneously correlated with the CHA_2_DS_2_-VASc score and optimal clinical conditions for surgery. Among these, miR-22-3p, miR-128-3p, miR-130a-3p, miR-140-5p, miR-143-3p, miR-148b-3p, miR-497-5p, and miR-652-3p correlated positively with the score and negatively with the conditions for ablation, while miR-144-5p, miR-192-5p, and miR-194-5p were negatively correlated with the CHA_2_DS_2_-VASc score and positively correlated with surgical suitability (48). It is yet to be confirmed whether any of these miRNAs, which do not have been reported as cardiac-enriched miRNAs (49), can be transferred to clinical practice to establish the risk of stroke in patients who could benefit from catheter ablation.

In a most recent report, Liu et al. studied the serum levels of miR-106 (and its target *MYL4*) in relation to the prevalence, risk stratification, and prognosis in a case/control study of 300 AF patients and 300 controls (50). Findings from this study suggested that levels of miR-106 closely related with the severity and prevalence of AF, meanwhile, a positive and significant correlation was also found between the risk from CHA_2_DS_2_ score and the levels of miR-106. Interestingly, these authors found a higher miR-106 expression in the right atrial appendage of AF patients *vs.* tissue from patients with sinus rhythm which suggests that this miRNA has a direct participation in the endpoints that the authors analyzed (50). However, it is necessary to verify the functional role of miR-106 in cardiomyocytes with additional trials conducted to provide a strong basis confirming that its circulating levels are a reliable biomarker for MACE risk in AF patients.

miRNAs are also released into circulation by using several strategies that guarantee their delivery in organs and tissues where remote functions will be carried out. As packaging systems, miRNAs have been described as wrapped in: (i) vesicles, mainly exosomes (30–100 nm in size) and microvesicles (50–1,000 nm) (51, 52); (ii) bound to proteins, such as AGO2 or high density lipoproteins (HDL) (53, 54), and (iii) apoptotic bodies (4 μm) (55). Indeed, several groups have described differences in the levels of circulating extracellular vesicles (EVs) between patients with and without AF (56, 57). On the other hand, these EVs have been reported to be procoagulant (58, 59). In this context, the main cause of stroke in the general population has been described to be the embolization of the thrombus typically formed in the left atrial appendage in the context of AF (60). The relationship between AF, EVs, miRNAs, and the risk of stroke has recently been tested by Zietzer et al. (61). These authors characterized cell-derived EVs (endothelial cells, platelets, and erythrocytes) isolated from the left atrial appendage of patients with permanent AF, non-permanent AF, and no history of AF. Their results showed a significantly higher accumulation of EVs specifically derived from platelets in the left atrial appendage in permanent *vs*. non-permanent AF. Based on *in vitro* experiments, these authors suggested that, in a state of platelet activation, platelet-derived EVs loaded with miR-223 transfer this miRNA to endothelial cells, thereby reducing endothelial healing (61), a key process to control thrombosis (62). These data therefore suggest for the first time a role for EVs loaded with miRNAs as a thrombogenic mechanism in AF. Although they should be confirmed in other series of patients and more in-depth basic studies are necessary, they may constitute an interesting starting point for future research on the role of miRNAs carried by EVs in MACE in patients with AF.

## 6. miRNAs as markers of immunothrombosis in AF

The previously reviewed studies place miRNAs only as descriptive markers of risk of MACE in AF, since they associate circulating levels of miRNAs with the occurrence of events, but they do not investigate the mechanistic basis of such association. In this section, in which the thromboinflammatory component of AF will be reviewed, we will describe a few studies that delve into the molecular mechanisms that could explain the implication of miRNAs in an increased risk of MACE in AF.

As briefly mentioned before, the link between CVD, inflammation, and thrombosis is supported by the prothrombotic consequences of endothelial dysfunction and platelet activation promoted by the activation of inflammatory cells and their mediators (9). The CANTOS trial demonstrated the clinical usefulness of canakinumab (which targets the IL-6β pathway) to diminish the recurrence of cardiovascular events in patients with previous AMI (63). In the case of AF, the susceptibility to thrombosis persists despite anticoagulation prophylaxis with anti-vitamin K, which are drugs that reduce thrombin generation (64) rather than inflammation. Our group reported for the first time that the inflammatory background underlying the risk of MACE in AF is associated with one miRNA, miR-146a, a physiological brake of NF-κB inflammatory pathway (65). Thus, in 2014, our group described the role of a functional miR-SNP of miR-146a, rs2431697, as a predictor of MACE in 901 patients with AF (66). This miR-SNP transcriptionally regulates miR-146a levels, so carriers of T allele (MAF = 0.27) have only about 50% of mature miRNA compared with homozygous CC carriers (67). Our results demonstrated that rs2431697 genotype was not related to the occurrence of AF, but when it was added to the CHA_2_DS_2_-VASc + IL6, the accuracy of risk prediction was significantly increased compared to this scale alone. Furthermore, we demonstrated that stimulation of monocytes from healthy volunteers with LPS increased miR-146a levels while decreasing those of *IL6*, in a magnitude that was dependent on the rs2431697 genotype. In conclusion, we described a potent inflammatory element, miR-146a levels through rs2431697 genotype, as a prognostic marker for MACE in anticoagulated patients with AF (66).

At that time, the term immunothrombosis was coined by Engelmann and Massberg to describe a unique process involving innate immunity and thrombosis, a physiological interaction which aims to protect organisms from pathogens (68). During this process, neutrophils get activated and release their cellular content in the form of neutrophil extracellular traps (NETs) in a process named NETosis. Those NETs contain different proteins such as histones and enzymes attached to a DNA backbone that aim to trap and inactivate pathogens. When immunothrombosis is overwhelmed thromboinflammation occurs, since neutrophils content activate proinflammatory and prothrombotic pathways (69). Thus, thromboinflammation has recently been recognized as a relevant factor in cardiovascular diseases (70). The functional role of miRNAs in thromboinflammatory processes is also being revealed, and their implication in NETosis and in cardiovascular diseases is a topic that is gaining interest (71). As we will detail below, in completing the circle formed by inflammation, innate immunity, and thrombosis our group went one step further, characterizing for the first time the participation of a miRNA, miR-146a, in immunothrombosis through NET formation (72).

Several studies have shown that components of NETs are inflammatory and prognostic markers in various cardiac diseases, including AF (73–76). In turn, the Rotterdam Study has recently investigated the relationship between some immunothrombotic elements and new-onset AF in several thousand patients, but no independent association between the selected biomarkers and the disease was found (77). Even so, these authors sustained that the link between immunothrombosis and AF still occurs, probably mediated by other CV factors co-exiting in naive AF patients. In relation to patients with already established AF, our group demonstrated that neutrophil elastase (a subrogated marker of NETosis) was an independent marker of increased risk of CV events, CV mortality, and all-cause mortality in these patients (78). The predictive ability of all-cause mortality, CV mortality, CV events, and ischemic stroke was slightly higher when elastase levels were added, not being this sum, however, superior to CHA_2_DS_2_-VASc score. In addition, we provided molecular insights into the relationship between rs2431697 and MACEs. Results from *in vitro* approaches determined that miR-146a deficiency establishes that a lower inflammatory threshold is necessary to activate NETosis. Moreover, our results from WT and miR-146a^–/–^ mice models suggested a causal relationship between miR-146a and NETosis (78). Overall these data suggested that NETosis coexists with the inflammation underlying AF and further, that miR-146 adds to this background by modulating the risk of MACE in these patients. However, these results need to be further confirmed in future studies, to determine if either or both (surrogate NETs markers and/or the miR-146a rs2431697 genotype) are reliable prognostic markers of MACE in patients with AF.

## 7. Conclusion and perspectives

The emergence of miRNAs as important players in the pathophysiology of AF and, as shown in the present review, in the development of MACE in AF has added a new layer in the understanding of this disease (Table 1). However, several points need further investigation and must be implemented. The first point is the standardization of the use of miRNAs as biomarkers in any disease. There are still many inconsistencies in the measurement of miRNAs in biofluids that biased many studies. The main effort must be carried out in the methods to obtain RNA. Recently, Mussbacher et al. have shown that the anticoagulant used for blood extraction, time until RNA obtention, and storage temperature were important to obtain reproducible results (79). Other basic tests must also be performed, such as hemolysis evaluation (80). RNA extraction method is also an important point that needs standardization since it can condition the integrity of the RNA and DNA contamination (81). The size of the cohort used for the discovery initial step is also of paramount importance. Searching for differentially expressed miRNAs in biofluids using small cohorts leads to confounding results (82). Thus, an important effort should be made in order to standardize the methodology concerning the use of miRNAs (and other ncRNAs) as biomarkers to go forward and include these molecules as an additional tool in personalized medicine. Indeed, this would allow a more accurate risk stratification, diagnosis, and prognosis for MACE in AF.

**TABLE 1:** Summary of mentioned miRNAs with a potential role in predicting major adverse cardiovascular events (MACE) in atrial fibrillation patients.

miRNAs	Expression	Function	References
miR-22-3p	Higher plasma levels	↑↑ Risk of MACE	(46)
miR-107	Higher plasma levels	↑↑ Risk of MACE	(46)
		Improve the 2MACE score	
miR-146a-5p	Lower plasma levels	↑↑ Risk of MACE	(46)
		Improve the 2MACE score	
	rs2431697 genotype	Prognostic marker for MACE	(66, 78)
miR-128-3p, miR-130a-3p, miR-140-5p, miR-143-3p, miR-148b-3p, miR-497-5p, miR-652-3p	Higher plasma levels	Correlates + with the CHA_2_DS_2_-VASc score	(48)
		Correlates–with the catheter ablation	
miR-144-5p, miR-192-5p miR-194-5p	Lower plasma levels	Correlates–with the CHA_2_DS_2_-VASc score	(48)
		Correlates + with the catheter ablation	
miR-106	Higher serum levels	↑↑ Severity and prevalence of AF	(50)
miR-223	Higher levels in EVs	↓ Endothelial healing–possibly thrombogenic	(61)

Another important point is the potential use of miRNA mimics or inhibitors as new tools to fight CVD. The use of miR-146a has been successfully used in different animal models of CVD, demonstrating that injection of this miRNA reduces the onset of myocardial ischemia reperfusion injury (83), myocardial infarction (84), or atherosclerosis (85). Indeed, there are many preclinical and clinical trials that are on-going using miRNAs in different diseases (86). Different technical difficulties have to be overcome to allow the use of miRNAs in therapy. Among these, the search of stable molecules through chemical modifications will help to avoid their degradation by RNAses. The search of carriers to specifically bring miRNAs to their target organ/cell is also an important issue (87). At the moment, nanotechnology strategies such as nanoparticles and extracellular vesicles, or hydrogels are being tested (88).

In conclusion, miRNAs may be very helpful as a new clinical tool both as biomarkers or therapeutic tool to avoid the development of MACE in AF. However, the use of miRNAs in diagnosing and predicting MACE in AF has still a long way ahead and new studies have to be performed to better understand the functional role of miRNAs in the pathophysiology of CVD complications in AF and to determine their use as trustable biomarkers.

## Author contributions

All authors listed have made a substantial, direct, and intellectual contribution to the work, and approved it for publication.
